# Supplementary figures and images for: A biophysical model of striatal microcircuits suggests gamma and beta oscillations interleaved at delta/theta frequencies mediate periodicity in motor control
Source: PLoS Comput Biol. 2020 Feb 25;16(2):e1007300. doi: 10.1371/journal.pcbi.1007300 (PMC7059970; doi:10.1371/journal.pcbi.1007300)

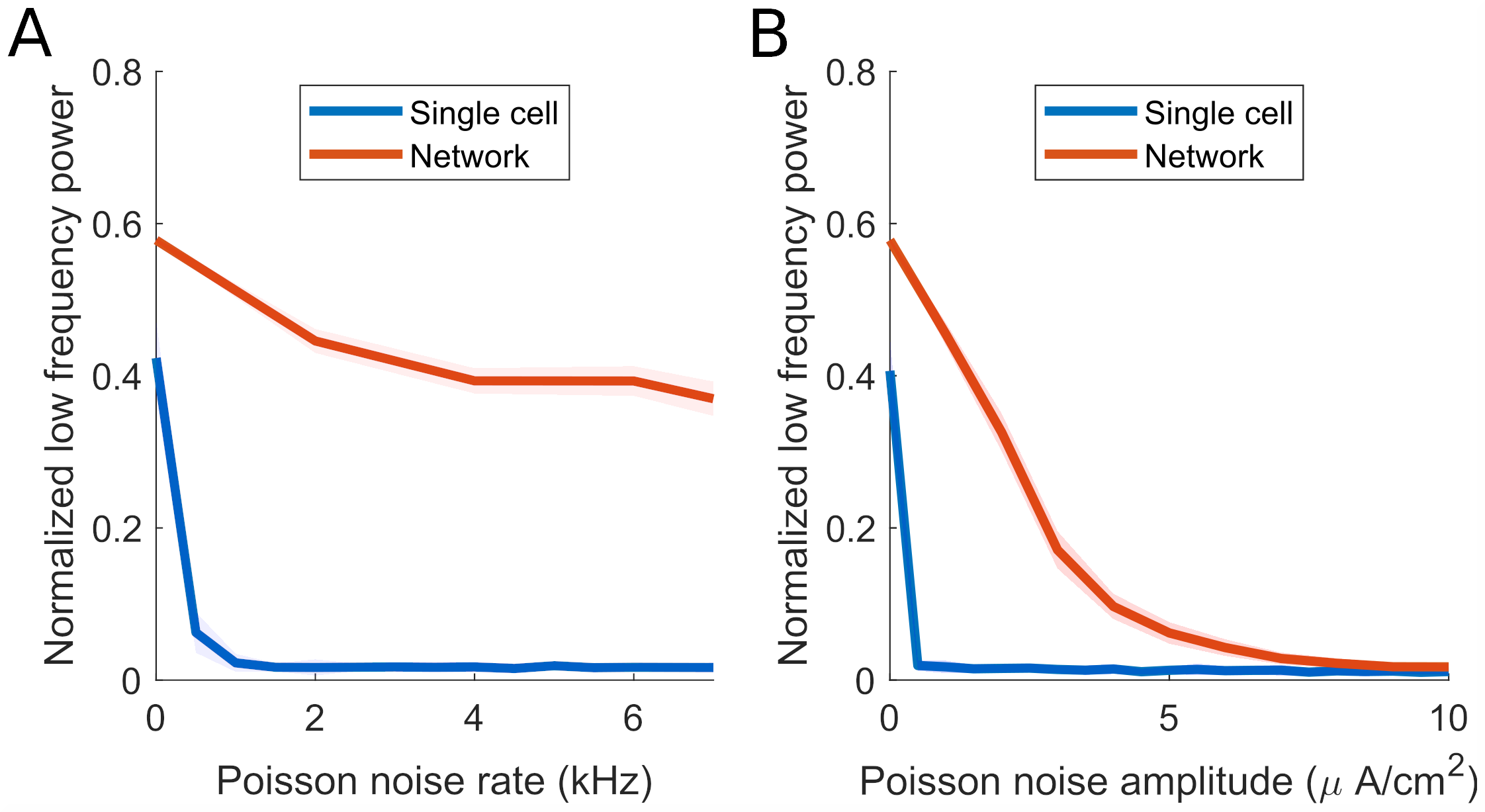

Supplement: S1 Fig — (A) Plot of normalized low frequency (<10 Hz) power of the voltage of a single model FSI (blue) and the summed voltages of the high DA FSI network (red) as Poisson noise of varying rate is applied. Each cell in the network receives the same amount of noise that the isolated cell receives. Iapp = 14 μA/cm2 for all simulations; in the high DA FSI network, ggap = 0.3 mS/cm2, gsyn = 0.005 mS/cm2. The solid line represents the mean value over 10 simulations per point. Shading represents standard deviation from these means. Power spectra are derived using Thomson’s multitaper power spectral density (PSD) estimate (MATLAB function pmtm). (B) Plot of normalized low frequency (<10 Hz) power of the voltage of a single model FSI and the summed voltages of the high DA FSI network as Poisson noise of varying amplitude is applied. (TIF) [file pcbi.1007300.s001.tif]

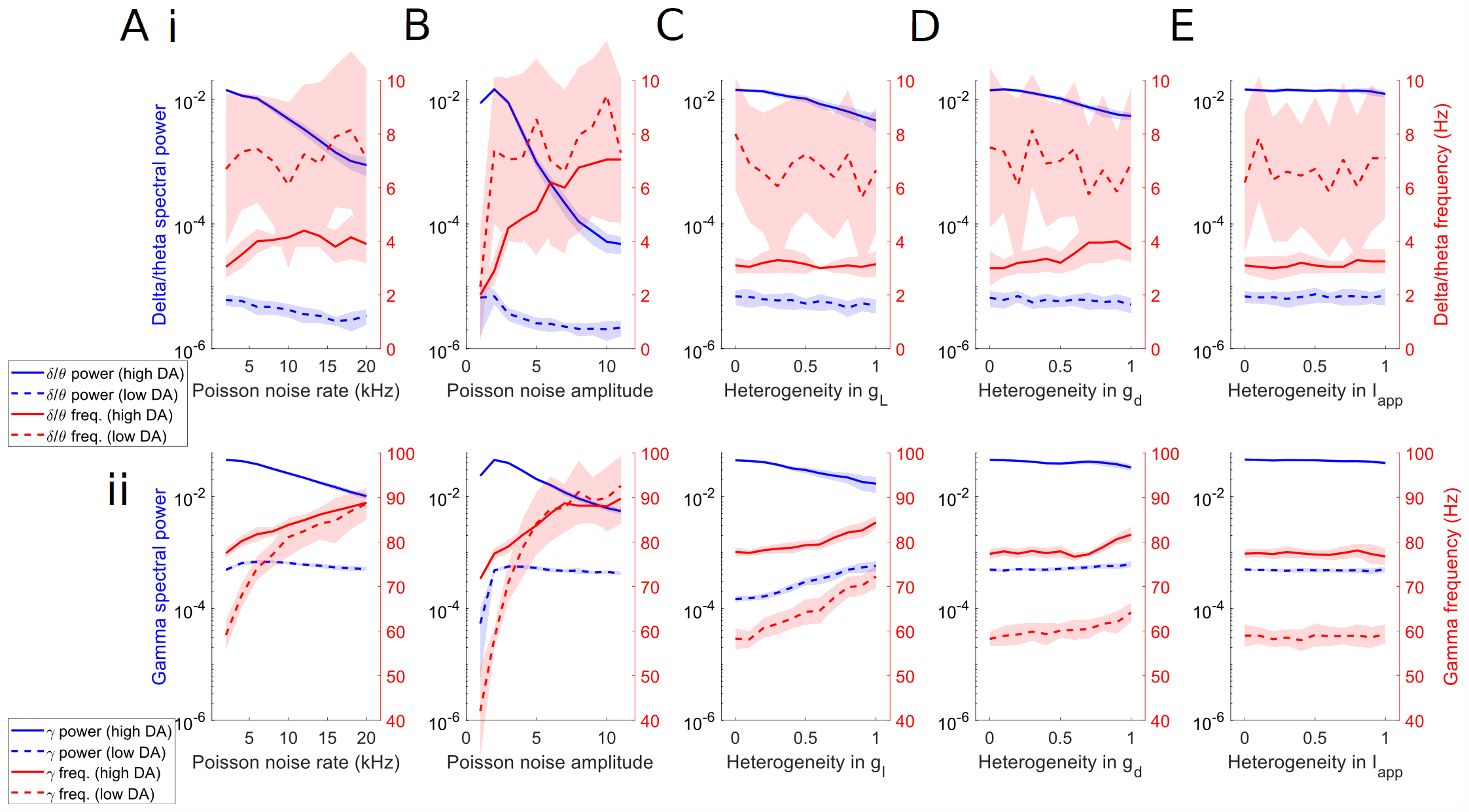

Supplement: S2 Fig — Power and frequency of δ/θ and γ rhythms in FSI network mean voltage as a function of (A) noise frequency, (B) noise amplitude, (C) heterogeneity in leak current conductance, (D) heterogeneity in potassium D current conductance, and (E) heterogeneity in applied current. For heterogeneity values, 0 represents completely uniform values and 1 represents a level of heterogeneity where values vary between zero and twice the default value. Default leak current conductance is 0.25 mS/cm2 and default D current conductance is 6 mS/cm2; default applied current is 7 mA/cm2 for low DA and 14 mA/cm2 for high DA. The parameters not being varied in plots A-C are held at either the high DA values (solid lines, Iapp = 14 μA/cm2, ggap = 0.3 mS/cm2, gsyn = 0.005 mS/cm2) or the low DA values (dotted lines, Iapp = 7 μA/cm2, ggap = 0.15 mS/cm2, gsyn = 0.1 mS/cm2), according to the legend. The solid line represents the mean value over 10 simulations per point. Shading represents standard deviation from these means. Power spectra are derived using Thomson’s multitaper power spectral density (PSD) estimate (MATLAB function pmtm). (TIF) [file pcbi.1007300.s002.tif]

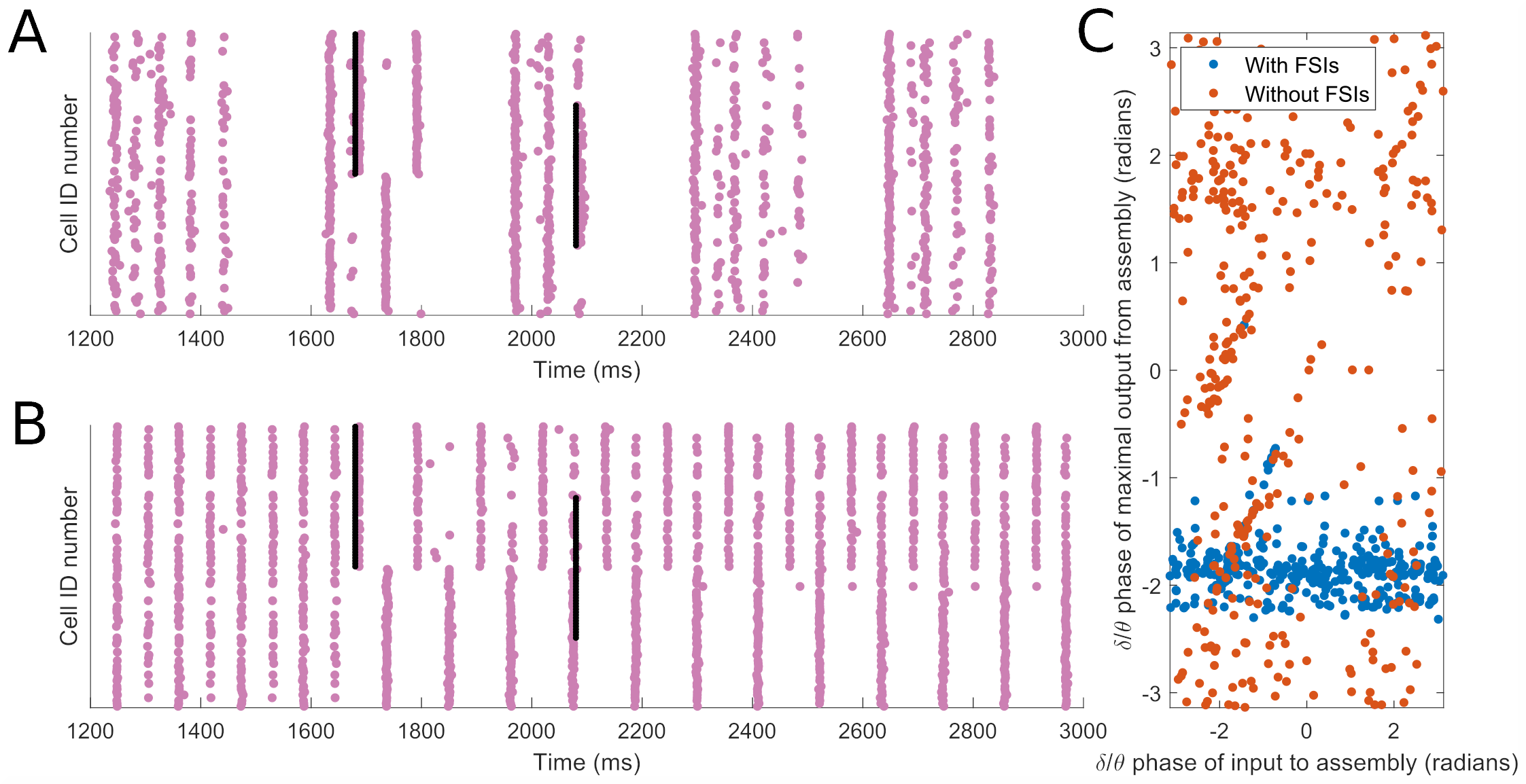

Supplement: S3 Fig — (A) Example raster plot of the D1 SPN subnetwork receiving δ/θ frequency FSI input while being subjected to input during high DAergic tone: An excitatory 20 millisecond pulse of input is provided to cells 50-100 (assembly 1) at t = 1680 ms and a later excitatory pulse of input is provided to cells 25-75 (assembly 2) at t = 2080 ms. Assembly 1 is active for several β cycles after the first input, causing rebound spiking at antiphase of the cells not in assembly 1 (as in McCarthy 2011 [45]), but becomes inactive during the δ/θ peak beginning around t = 1800 ms. Assembly 2 can then respond with a high degree of coherence shortly after the second input. (B) Example raster plot of the isolated D1 SPN subnetwork (not receiving any FSI input) being subjected to the input during high DAergic tone. The same two excitatory pulses are provided. Assembly 1 and its antiphase activity begin firing similarly to the example in (A), but since there is no δ/θ input, the β-rhythm firing of assembly 1 persists indefinitely. Input to assembly 2 is thereby unable to generate a specific response, and the coherence of assembly 1 persists even after the second input. (C) Plot showing history-independence of SPN responses when FSIs are present. Regardless of the phase at which input is given, the maximal response of SPNs in any given cell assembly occurs at a preferred δ/θ phase around -2 radians, “erasing” the information of when the input arrived. When FSIs are not present, there is no theta rhythm in the network, and the response of the cells to input is more random. (TIF) [file pcbi.1007300.s003.tif]

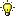

Supplement: S1 File — (ZIP) [file pcbi.1007300.s004.zip › striatum-standalone/dynasim/functions/dependencies/m2html/templates/3frames/demoicon.gif]

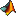

Supplement: S1 File — (ZIP) [file pcbi.1007300.s004.zip › striatum-standalone/dynasim/functions/dependencies/m2html/templates/3frames/matlabicon.gif]

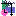

Supplement: S1 File — (ZIP) [file pcbi.1007300.s004.zip › striatum-standalone/dynasim/functions/dependencies/m2html/templates/3frames/simulinkicon.gif]
