# Supplementary material for: A biophysical model of striatal microcircuits suggests gamma and beta oscillations interleaved at delta/theta frequencies mediate periodicity in motor control
Source: PLoS Comput Biol. 2020 Feb 25;16(2):e1007300. doi: 10.1371/journal.pcbi.1007300 (PMC7059970; doi:10.1371/journal.pcbi.1007300)
Supplement: S1 File — (ZIP) [file pcbi.1007300.s004.zip › striatum-standalone/dynasim/functions/dependencies/m2html/templates/3frames/mdir.tpl]

Index for Directory {MDIR}


# Index for directory "{MDIR}"

## Matlab files in this directory:

- {NAME}     -  {H1LINE}


## Other Matlab-specific files in this directory:

- {OTHERFILE}


## Top directories:

- Toplevel directory
- Main directory

## Subsequent directories:

- {SUBDIRECTORY}


## Dependency Graph

- View the Graph.


## TODO List

- View the TODO list.


---

Generated on {DATE} by **m2html** using template **3frames** © Guillaume Flandin, Lorenz Gerstmayr, 2003-2005
