# Supplementary material for: A biophysical model of striatal microcircuits suggests gamma and beta oscillations interleaved at delta/theta frequencies mediate periodicity in motor control
Source: PLoS Comput Biol. 2020 Feb 25;16(2):e1007300. doi: 10.1371/journal.pcbi.1007300 (PMC7059970; doi:10.1371/journal.pcbi.1007300)
Supplement: S1 File — (ZIP) [file pcbi.1007300.s004.zip › striatum-standalone/dynasim/functions/dependencies/m2html/templates/blue/master.tpl]

Matlab Index


# Matlab Index

## Matlab Directories

- {DIR}

## Matlab Files found in these Directories

|  |
| --- |
| {IDNAME} |

## Search Engine

Search for 


## Dependency Graph

- View the Graph.


---

Generated on {DATE} by **m2html** © 2005
