# Supplementary material for: A biophysical model of striatal microcircuits suggests gamma and beta oscillations interleaved at delta/theta frequencies mediate periodicity in motor control
Source: PLoS Comput Biol. 2020 Feb 25;16(2):e1007300. doi: 10.1371/journal.pcbi.1007300 (PMC7059970; doi:10.1371/journal.pcbi.1007300)
Supplement: S1 File — (ZIP) [file pcbi.1007300.s004.zip › striatum-standalone/dynasim/functions/dependencies/m2html/templates/brain/graph.tpl]

Dependency Graph for {MDIR}


# Dependency Graph for {MDIR}

{GRAPH\_MAP}


---

Generated by **m2html** © 2005
