# Supplementary material for: A biophysical model of striatal microcircuits suggests gamma and beta oscillations interleaved at delta/theta frequencies mediate periodicity in motor control
Source: PLoS Comput Biol. 2020 Feb 25;16(2):e1007300. doi: 10.1371/journal.pcbi.1007300 (PMC7059970; doi:10.1371/journal.pcbi.1007300)
Supplement: S1 File — (ZIP) [file pcbi.1007300.s004.zip › striatum-standalone/dynasim/functions/dependencies/m2html/templates/brain/mfile.tpl]

Description of {NAME}


# {NAME}

## PURPOSE

**{H1LINE}**

## SYNOPSIS

**{SYNOPSIS}  This is a script file.**

## DESCRIPTION

```
{DESCRIPTION}
```

## CROSS-REFERENCE INFORMATION

This function calls:

- {NAME\_CALL} {H1LINE\_CALL}

This function is called by:

- {NAME\_CALLED} {H1LINE\_CALLED}


## SUBFUNCTIONS

- {SUB}


## DOWNLOAD

{NAME}.m


## SOURCE CODE

```
{SOURCECODE}
```


---

Generated on {DATE} by **m2html** © 2005
