# Supplementary material for: A biophysical model of striatal microcircuits suggests gamma and beta oscillations interleaved at delta/theta frequencies mediate periodicity in motor control
Source: PLoS Comput Biol. 2020 Feb 25;16(2):e1007300. doi: 10.1371/journal.pcbi.1007300 (PMC7059970; doi:10.1371/journal.pcbi.1007300)
Supplement: S1 File — (ZIP) [file pcbi.1007300.s004.zip › striatum-standalone/dynasim/functions/dependencies/MDD_SM/html/demo_MDD.html]

Multidimensional Dictionary - Demo 

# Multidimensional Dictionary - Demo

File demonstrating some of the basic capabilities of MDD on a sample neuroscience dataset. For full instructions on using MDD, see tutorial\_MDD.m

## Contents

- Overview
- Set up paths and formatting
- Load a sample dataset and build an MDD object
- What is an MDD object and why use one?
- MDD subscripts and indexing
- Advanced indexing methods
- Running functions on MDD objects
- Plotting 3D data
- Modularization of plotting
- Merging two MDD objects
- Packing MDD dimensions (analogous to cell2mat)
- Unpacking MDD dimensions
- Next steps
- Summary so far

## Overview

MDD is a MATLAB tool for managing high-dimensional data that often arises in scientific data analysis. It can be thoughout of as a MATLAB cell array (or matrix) with some additional functionality.

## Set up paths and formatting

```
% Format
format compact
format short g

% Check if in MDD folder
if ~exist(fullfile('.','data','sample_data.mat'), 'file')
    error('Should be in MDD folder to run this code.')
end

% Add MDD toolbox to Matlab path if needed
if ~exist('MDD','class')
  addpath(genpath(pwd));
end
```

## Load a sample dataset and build an MDD object

```
% Load some sample simulated data
load('sample_data.mat');
load('sample_data_meta2.mat');
```

The file *sample\_data.mat* contains *dat*, a 4-dimensional cell array:

```
whos dat
```

```
  Name      Size                  Bytes  Class    Attributes

  dat       3x3x2x8            21633984  cell
```

Each cell in dat contains some neural time series data. For example

```
dat(1,1,2,1)
```

```
ans =
  1×1 cell array
    {1001×20 single}
```

Use this to build an MDD object.

```
% Construct MDD object
mdd = MDD(dat,axis_vals,axis_names);
```

Note that *axis\_vals* and *axis\_names* contain useful metadata, but their details are not important here.

## What is an MDD object and why use one?

When working with high-dimensional data, like that cell array *dat*, it's often difficult to keep track of what each dimension represents. **MDD objects provide a way of organizing and manipulating this information.**

Let's print a summary of the data contained within *mdd*:

```
% Print object summary
mdd.printAxisInfo
```

```
Axis Size: [3  3  2  8]
Axis 1: param1 (numeric) -> 0, 10, 20
Axis 2: param2 (numeric) -> 5, 10, 15
Axis 3: populations (cellstr) -> E, I
Axis 4: variables (cellstr) -> v, iNa_m, iNa_h, iK_n, I_iGABAa_s, E_iAMPA_s, I_iGABAa_ISYN, E_iAMPA_ISYN
```

This tells us several things about our MDD object:

- It is a 4-dimensional object with mdd.size = [3,3,2,8]
- The four axes are titled: param1, param2, populations, and variables
- This also summarizes the values that each axis takes on, and the corresponding data type of those values (numeric or cellstr). For example, the 'populations' axis takes on values 'E' and 'I' (for excitatory and inhibitory cells, respectively).

An MDD object can be thought of in several ways:

- A MATLAB matrix or cell array that can be indexed by using strings and regular expressions
- An N-dimensional table (a table is equivalent to an MDD object in 2-dimensions)
- A map/dictionary that associates multiple keys with a single value

## MDD subscripts and indexing

MDD contains several options for how to index data. The most basic method is to just index MDD objects like you would index normal Matlab matrices and cells. For example:

```
% Basic indexing
mdd3D = mdd(:,:,1,2:3);
mdd3D.printAxisInfo
```

```
Axis Size: [3  3  1  2]
Axis 1: param1 (numeric) -> 0, 10, 20
Axis 2: param2 (numeric) -> 5, 10, 15
Axis 3: populations (cellstr) -> E
Axis 4: variables (cellstr) -> iNa_m, iNa_h
```

This selects everything from dimensions 1 and 2, the 1st element from dimension 3, and the 2nd and 3rd elements from dimension 4. Another way to do this, however, is to reference the axis values directly. For example:

```
% Indexing by string query
mdd3D = mdd(:,:,'E','iNa');
mdd3D.printAxisInfo
```

```
Axis Size: [3  3  1  2]
Axis 1: param1 (numeric) -> 0, 10, 20
Axis 2: param2 (numeric) -> 5, 10, 15
Axis 3: populations (cellstr) -> E
Axis 4: variables (cellstr) -> iNa_m, iNa_h
```

Note that substring matching (based on Matlab's strcmp command) was used here to select both iNa\_m and iNa\_h. However, regular expressions can be used as well. The "/" denotes to use regular expressions for the search. This command picks out all variable names beginning with an "I".

```
% Indexing by regular expressions
mdd3D = mdd(:,:,'E','/^I/');
mdd3D.printAxisInfo
```

```
Axis Size: [3  3  1  2]
Axis 1: param1 (numeric) -> 0, 10, 20
Axis 2: param2 (numeric) -> 5, 10, 15
Axis 3: populations (cellstr) -> E
Axis 4: variables (cellstr) -> I_iGABAa_s, I_iGABAa_ISYN
```

If we only want to specify the values for a single axis, we can use axisSubset. This indexes the 'variables' axis for all values containing 'iNa'

```
% Indexing by axis-value pairs
mdd3D = mdd.axisSubset('variables', 'iNa');
mdd3D.printAxisInfo
```

```
Axis Size: [3  3  2  2]
Axis 1: param1 (numeric) -> 0, 10, 20
Axis 2: param2 (numeric) -> 5, 10, 15
Axis 3: populations (cellstr) -> E, I
Axis 4: variables (cellstr) -> iNa_m, iNa_h
```

All standard forms of Matlab indexing work as well, including indexing by position, linear indexing, and logical indexing.

```
% Method 1 - Indexing by position
foo1 = mdd(1:3,3,2,8);

% Method 2 - Linear indexing
foo2 = mdd(142:144);      % Take the last 3 entries in the data.

% Method 3 - Logical indexing
ind = false(1,144); ind(142:144) = true;
foo3 = mdd(ind);            % Using logical indexing also produces the same result.

disp(isequal(foo1,foo2,foo3));
clear foo1 foo2 foo3
```

```
   1
```

The results of all three indexing methods above are the same. For more details about indexing methods in Matlab, see: https://www.mathworks.com/help/matlab/math/array-indexing.html

## Advanced indexing methods

Finally, MDD supports inline queries using the valSubset method. Here are two examples.

```
% Select axis1 values greater than 5, and axis2 equal to 10
mdd3D = mdd.valSubset('>5','==10',:,:);
mdd3D.printAxisInfo
```

```
Axis Size: [2  1  2  8]
Axis 1: param1 (numeric) -> 10, 20
Axis 2: param2 (numeric) -> 10
Axis 3: populations (cellstr) -> E, I
Axis 4: variables (cellstr) -> v, iNa_m, iNa_h, iK_n, I_iGABAa_s, E_iAMPA_s, I_iGABAa_ISYN, E_iAMPA_ISYN
```

```
% Select axis1 values between 8 and 11, and axis2 values between 3 and 11
mdd3D = mdd.valSubset('8 < x < 11','3 < x <= 11',:,:);
mdd3D.printAxisInfo
```

```
Axis Size: [1  2  2  8]
Axis 1: param1 (numeric) -> 10
Axis 2: param2 (numeric) -> 5, 10
Axis 3: populations (cellstr) -> E, I
Axis 4: variables (cellstr) -> v, iNa_m, iNa_h, iK_n, I_iGABAa_s, E_iAMPA_s, I_iGABAa_ISYN, E_iAMPA_ISYN
```

## Running functions on MDD objects

The primary purpose of MDD is to make working with high-dimensional data more convenient. Traditionally, if you are working with an N-dimensional matrix/cell array, you would write a function that takes in that variable and knows what to do with each of the N dimensions (usually involving nested for loops). This function would not work on data with N-1 or N+1 dimensions.

MDD takes a different approach. With MDD, you specify function handles that operate on lower dimensional data (usually 1D, 2D, or 3D) and to "assign" these to specific dimension in your higher dimensional MDD object. The MDD method **recursiveFunc** is used for this. Examples below will show how recursiveFunc can be used for plotting. But it is not limited to this.

**Plotting setup**

This section can be safely ignored. Here I'm just adding a metadata structure to our mdd object, which will be used by some of the following plotting commands. This stores some additional metadata about the time series in *mdd*. I'm also defining a data structure defining how large to make the figures.

```
meta = struct;
meta.datainfo(1:2) = MDDAxis;
meta.datainfo(1).name = 'time(ms)';
meta.datainfo(1).values = time;
meta.datainfo(2).name = 'cells';
meta.datainfo(2).values = [];
mdd.meta = meta;
clear meta

op.figwidth=0.4;
op.figheight=0.3;
```

## Plotting 3D data

The workhorse of performing functions on MDD objects is **recursiveFunc**. Here is an example of how it is used to plot our data.

First, we will pull out a 3D subset of the data. Our data consists of 9 simulations of a neural network, in the form of a 3x3 parameter sweep (axes 1 and 2). For each simulation, we have data for the two different types of neurons simulated, excitatory (E) and inhibitory (I) cells. We also have the variables associated with each neuron (for example, membrane voltage, ionic currents, etc.). Here, we will only look at the voltage of the neurons.

```
% Pull out a 3D MDD object with only E cell membrane voltage
mdd3D = mdd(:,1:2,:,'v');
mdd3D.printAxisInfo
```

```
Axis Size: [3  2  2  1]
Axis 1: param1 (numeric) -> 0, 10, 20
Axis 2: param2 (numeric) -> 5, 10
Axis 3: populations (cellstr) -> E, I
Axis 4: variables (cellstr) -> v
```

Now, let's run the plot:

```
% Set up plotting arguments
function_handles = {@xp_handles_fignew,@xp_subplot_grid,@xp_matrix_basicplot};
dimensions = {{'populations'},{'param1','param2'},{'data'}};
function_arguments = {{op},{},{}};

% Run the plot. Note the "+" icons next to each plot allow zooming.
close all
mdd3D.recursiveFunc(function_handles,dimensions,function_arguments);
```

To break down what's going on here:

- *function\_handles* - A cell array of function handles that specify what to do with specific dimensions of the data.
- *dimensions* - A cell array that tells which dimensions of the data should be assigned to which function handles.
- *function\_arguments* - A cell array that tells what arguments to pass to each of the function handles. Here we pass *op*, which tells xp\_handles\_fignew how big to make the figure.
- There should be 1 entry in *dimensions* and *function\_arguments* for every function handle supplied. (E.g., their lengths should be equal)

In this example, the 'populations' dimension of our data (either E cells or I cells) is handled by the function *xp\_handles\_fignew*, which creates a new figure for each value along this dimension provided. The dimensions 'param1' and 'param2' are handled by xp\_subplot\_grid, which creates a grid of subplots for the parameter sweep. Note that xp\_subplot\_grid operates on 2D data. Finally, xp\_matrix\_basicplot creates the actual plots, operating on the time series data contained in *mdd.data*

Here is the result:

 

Note that each of the functions passed to *function\_handles* is essentially acting on lower-dimensional MDD objects. For example, xp\_matrix\_basicplot operates on 0D data (e.g., a single cell)

```
% Call xp_matrix_basicplot directly with 0D data (e.g.,
foo = mdd(1,1,2,1);
close all
figure; xp_matrix_basicplot(foo);
```

Similarly, xp\_subplot\_grid operates on 2D data, but it can only sets up the subplots and cannot plot anything. It needs recursiveFunc to chain everything together.

## Modularization of plotting

The advantage of recursiveFunc is that the function handles we use can be recycled and applied in different contexts. This allows us to work with data of different dimensionality and to slice the data in different ways. Below are two additional examples of this.

**2D plot, sliced to visualize populations vs variables**

Here we will use similar methods to look at our data in a different way. We will look at a single simulation (param values), and comparing both E and I cells and their underlying variables

```
% Pull out a 2D MDD object (variables axis is indexed using regular expressions)
mdd2D = mdd(2,2,:,'/v|iNa|iK/');
mdd2D.printAxisInfo

% Set up plotting arguments
function_handles = {@xp_subplot_grid,@xp_matrix_basicplot};
dimensions = {{'variables','populations'},{'data'}};
function_arguments = {{},{}};

% Run the plot
close all
figure; mdd2D.recursiveFunc(function_handles,dimensions,function_arguments);
```

```
Axis Size: [1  1  2  4]
Axis 1: param1 (numeric) -> 10
Axis 2: param2 (numeric) -> 10
Axis 3: populations (cellstr) -> E, I
Axis 4: variables (cellstr) -> v, iNa_m, iNa_h, iK_n
```

**2D plot, sliced to visualize populations vs param1**

Here we will repeat our above 3D plot, but instead group populations and param1 into the same set of subplots, and ignore param2. We also swapped in xp\_matrix\_imagesc for xp\_matrix\_basicplot, which represents the data using imagesc plots.

```
% Pull out a 2D MDD object
mdd2D = mdd(:,2,:,'v');
mdd2D.printAxisInfo

% Set up plotting arguments
function_handles = {@xp_subplot_grid,@xp_matrix_imagesc};
dimensions = {{'populations','param1'},{'data'}};
function_arguments = {{},{}};

% Run the plot
close all
figure; mdd2D.recursiveFunc(function_handles,dimensions,function_arguments);
```

```
Axis Size: [3  1  2  1]
Axis 1: param1 (numeric) -> 0, 10, 20
Axis 2: param2 (numeric) -> 10
Axis 3: populations (cellstr) -> E, I
Axis 4: variables (cellstr) -> v
```

## Merging two MDD objects

MDD also contains methods to perform operations on MDD objects. Here is an example of how two MDD objects can be merged together. For more details on methods like merge, see tutorial\_MDD.m

```
% Slice the dataset one way
close all
mdd1 = mdd(2,:,'E','v');

% Slice it another way
mdd2 = mdd(:,3,'E','v');
```

Notice that mdd1 and mdd2 are overlapping at (2,3,1,1). Hence, when we merge, we will set forceMergeBool to true. Without this, it will throw a warning

```
% Merge with overwrite
mdd_merged = merge(mdd1,mdd2,true);
```

Now, plot the merged data

```
dimensions = {[1,2],0};
close all; figure; recursiveFunc(mdd_merged,{@xp_subplot_grid,@xp_matrix_imagesc},dimensions);
```

Compare to the original dataset

```
close all; figure; recursiveFunc(mdd(:,:,'E','v'),{@xp_subplot_grid,@xp_matrix_imagesc},dimensions);
```

## Packing MDD dimensions (analogous to cell2mat)

MDD object properties include the raw data (usually in the form of a cell array) plus associated metadata. The raw data is an accessible property of *mdd*:

```
% Take a slice of mdd
mdd2 = mdd(:,:,:,1);

% View MDD data
mdd2.data
```

```
  3×3×2 cell array
ans(:,:,1) = 
    {1001×80 single}    {1001×80 single}    {1001×80 single}
    {1001×80 single}    {1001×80 single}    {1001×80 single}
    {1001×80 single}    {1001×80 single}    {1001×80 single}
ans(:,:,2) = 
    {1001×20 single}    {1001×20 single}    {1001×20 single}
    {1001×20 single}    {1001×20 single}    {1001×20 single}
    {1001×20 single}    {1001×20 single}    {1001×20 single}
```

There are several basic MDD operations for acting on data within an MDD object. *packDim* allows takes a dimension from the MDD object and pushes it into the internal *data* property. Here we will use it to pack the two cell populations (E and I) into *mdd.data*, so that they can be plotted simultaneously.

```
% First, let's average dimension 2 for every cell in _mdd2.data_. This
% averages all the traces together.
mdd2.data = cellfun(@(x) squeeze(mean(x,2)), mdd2.data, 'UniformOutput', false);
mdd2.data
```

```
  3×3×2 cell array
ans(:,:,1) = 
    {1001×1 single}    {1001×1 single}    {1001×1 single}
    {1001×1 single}    {1001×1 single}    {1001×1 single}
    {1001×1 single}    {1001×1 single}    {1001×1 single}
ans(:,:,2) = 
    {1001×1 single}    {1001×1 single}    {1001×1 single}
    {1001×1 single}    {1001×1 single}    {1001×1 single}
    {1001×1 single}    {1001×1 single}    {1001×1 single}
```

Now, pack the 'populations' dimension into *mdd2.data*

```
% Pack populations into mdd2.data
mdd2 = mdd2.packDim('populations');
mdd2.printAxisInfo
```

```
Axis Size: [3  3  1  1]
Axis 1: param1 (numeric) -> 0, 10, 20
Axis 2: param2 (numeric) -> 5, 10, 15
Axis 3: Dim 3 (numeric) -> 1
Axis 4: variables (cellstr) -> v
```

The populations property is now missing from mdd2. Instead, mdd2.data contains this information - it is now 1001x2 instead of 10001x1

```
% View the contents of mdd2.data
mdd2.data
```

```
ans =
  3×3 cell array
    {1001×2 single}    {1001×2 single}    {1001×2 single}
    {1001×2 single}    {1001×2 single}    {1001×2 single}
    {1001×2 single}    {1001×2 single}    {1001×2 single}
```

Plot the result

```
close all
figure; mdd2.recursiveFunc({@xp_subplot_grid,@xp_matrix_basicplot},{{'param1','param2'},{'data'}});
```

Here, red traces are "I" cells and blue traces are "E" cells.

## Unpacking MDD dimensions

Conversely, MDD dimensions can also be unpacked. Here, we will use the unpackDim method to pull out individual traces and select a few to examine more closely.

```
% Take a slice of mdd (variables axis is indexed using regular expressions)
mdd2 = mdd(2,2,'E','/v|iNa|iK/');

% Unpack the 2nd dimension from mdd.data (corresponding to individual
% traces) and create a new, 5th dimension of mdd to store this.
src = 2; dest = 5;
mdd2=mdd2.unpackDim(src, dest);
mdd2.printAxisInfo
```

```
Axis Size: [1   1   1   4  80]
Axis 1: param1 (numeric) -> 10
Axis 2: param2 (numeric) -> 10
Axis 3: populations (cellstr) -> E
Axis 4: variables (cellstr) -> v, iNa_m, iNa_h, iK_n
Axis 5: matrix_dim_2 (numeric) -> 1, 2, 3, 4, 5, 6, 7, 8, 9, 10, ...
```

We now have a 5th dimension of *mdd2* corresponding to each of the 80 traces that was originally in *mdd2.data*. Each of these tracs corresponds to a single cell in the simulation, so we can look at these more closely. Let's select a few.

```
% Select a few cells. Note we are also renaming axis5.
mdd2.axis(5).name = 'Cell Number';
mdd2 = mdd2(:,:,:,:,[1,3,6,9]);
mdd2.printAxisInfo
```

For more details on how to rename axes, see tutorial\_MDD.m

```
Axis Size: [1  1  1  4  4]
Axis 1: param1 (numeric) -> 10
Axis 2: param2 (numeric) -> 10
Axis 3: populations (cellstr) -> E
Axis 4: variables (cellstr) -> v, iNa_m, iNa_h, iK_n
Axis 5: Cell Number (numeric) -> 1, 3, 6, 9
```

Finally, plot the result

```
% Plot using recursiveFunc
close all
figure; mdd2.recursiveFunc({@xp_subplot_grid,@xp_matrix_basicplot},{{'variables','Cell Number'},{'data'}});
```

## Next steps

To start using MDD yourself, see `tutorial\_MDD.m`. In particular, this will provide additional details on how to build your own MDD objects and advanced methods to manipulate them.

```
% Run the following
edit tutorial_MDD.m
```

## Summary so far

At its core, MDD extends the way cells and matrices are indexed by allowing string labels to be assigned to each dimension of the cell array, similar to how row and column names are assigned to a table (e.g., in Pandas or SQL). MDD objects can then be indexed, sorted, merged, and manipulated according to these labels. (Section: MDD subscripts and indexing)

Additionally, MDD includes methods for performing operations on high dimensional data. The goal is to modularize the process of working with high dimensional data. Within MDD, functions designed to work on low dimensional data (1 or 2 dimension) can each be assigned to each operate on different dimensions of a higher dimensional object. Chaining several of these functions together can allow the entire high dimensional object to be processed. The advantage of this modular approach is that functions can be easily assigned other dimensions or swapped out entirely, without necessitating substantial code re-writes. (Section: Running functions on MDD objects)

Published with MATLAB® R2018b
