# Supplementary material for: A biophysical model of striatal microcircuits suggests gamma and beta oscillations interleaved at delta/theta frequencies mediate periodicity in motor control
Source: PLoS Comput Biol. 2020 Feb 25;16(2):e1007300. doi: 10.1371/journal.pcbi.1007300 (PMC7059970; doi:10.1371/journal.pcbi.1007300)
Supplement: S1 File — (ZIP) [file pcbi.1007300.s004.zip › striatum-standalone/dynasim/functions/dependencies/MDD_SM/html/demo_MDD_simple.html]

Multidimensional Dictionary - Simple Demo 

# Multidimensional Dictionary - Simple Demo

File demonstrating some the basic capabilities of MDD. For full instructions on using MDD, see tutorial\_MDD.m

## Contents

- Construct MDD object
- Indexing based on numerical query
- Indexing based on regular expression
- Merging MDD objects

## Construct MDD object

Enter some stock prices and create an MDD object

```
% Add MDD toolbox to Matlab path if needed
if ~exist('MDD','class')
  addpath(genpath(pwd));
end

% Some stock prices
stocks_data = [120.1, 175.3, 191.3;
    62.6, 90.3, 117.2;
    82.2 ,78.4 , 56.4;
    68.3,100.1, 98.2];

% Create mdd object
axis_labels= {{'Tech_Apple','Tech_Microsoft','Retail_CVS','Retail_Walmart'},2017:2019};
mdd = MDD(stocks_data,axis_labels);
```

This creates the following object:

```
% mdd =
%                         2017         2018         2019
% --------------------------------------------------------
% 'Tech_Apple'        |    120.1       175.3       191.3
% 'Tech_Microsoft'    |     62.6        90.3       117.2
% 'Retail_CVS'        |     82.2        78.4        56.4
% 'Retail_Walmart'    |     68.3       100.1        98.2
```

## Indexing based on numerical query

```
mdd2 = mdd(:,'2017 < x < 2019');
```

This returns the following:

```
% mdd2 =
%                         2018
% --------------------------------------------------------
% 'Tech_Apple'        |    175.3
% 'Tech_Microsoft'    |     90.3
% 'Retail_CVS'        |     78.4
% 'Retail_Walmart'    |    100.1
```

## Indexing based on regular expression

```
mdd3 = mdd('Tech_',:);
```

This returns the following:

```
% mdd3 =
%                         2017         2018         2019
% --------------------------------------------------------
% 'Tech_Apple'        |    120.1       175.3       191.3
% 'Tech_Microsoft'    |     62.6        90.3       117.2
```

## Merging MDD objects

The merge method allows mdd objects to be combined based on their axis labels.

```
% Merge mdd2 and mdd3
mdd_merged = merge(mdd2,mdd3,true);
mdd_merged.printAxisInfo
mdd_merged.data
```

This returns the following:

```
% mdd_merged =
%                         2017         2018         2019
% --------------------------------------------------------
% 'Tech_Apple'        |  [120.1]     [175.3]       [191.3]
% 'Tech_Microsoft'    |   [62.6]      [90.3]       [117.2]
% 'Retail_CVS'        |       []          []        [56.4]
% 'Retail_Walmart'    |       []          []        [98.2]
```

```
Axis Size: [4  3]
Axis 1: Dim 1 (cellstr) -> Tech_Apple, Tech_Microsoft, Retail_CVS, Retail_Walmart
Axis 2: Dim 2 (numeric) -> 2017, 2018, 2019
ans =
  4×3 cell array
    {[   120.1]}    {[175.3]}    {[   191.3]}
    {[    62.6]}    {[ 90.3]}    {[   117.2]}
    {0×0 double}    {[ 78.4]}    {0×0 double}
    {0×0 double}    {[100.1]}    {0×0 double}
```

Published with MATLAB® R2018b
