# Supplementary material for: A biophysical model of striatal microcircuits suggests gamma and beta oscillations interleaved at delta/theta frequencies mediate periodicity in motor control
Source: PLoS Comput Biol. 2020 Feb 25;16(2):e1007300. doi: 10.1371/journal.pcbi.1007300 (PMC7059970; doi:10.1371/journal.pcbi.1007300)
Supplement: Attachment — Submitted filename: response to reviewers.docx [file pcbi.1007300.s005.docx]

Reviewer #1: General comment: the Authors propose a novel model of striatal microcircuitry that explains how fast-spiking interneurons (FSIs) are key in allowing flexibility in motor programs, by modulating the dynamic oscillatory behavior in the network. While this work is relevant for the research in the field and generates interesting predictions, the results and, more importantly, the figures could be made more readable. Some examples are provided below.

On the other hand, the Authors made a great effort in discussing their findings, interpreting them in the context of motor behavior and diseases and highlighting the limitations of the model. One of the main limitation is the choice of the conductance value of the D-current for which, as the Authors acknowledge, there is little experimental evidence. The choice of this value is key for the results presented here.

- We thank the reviewers for their kind words and helpful comments. Each comment is addressed individually below.

Data availability: the Authors made the source code of their models publicly available on Github (<https://github.com/jchartove/striatum>), in agreement with the Journal policies, but no explanation is provided. In order to make the findings easily reproducible, it is of major importance that the source code is accompanied by an explanation on how to run the different scripts, which figure(s) could be easily reproduced, etc... (= add a readme file and description).

- A readme file describing how to run the simulations and replicate each of the figures in the text has been added, and additional documentation has been added to the runfiles and mechanism files for clarity.

The abstract (340 words) exceeds the maximum number of words (which is 300). The Authors could, for instance, move the detailed explanation of the model to the introduction.

- The abstract has been edited to be under the word limit.

Lines 12-13: delta oscillations could be mentioned here, because they appear later in the next without previous introduction (line 77).

- A reference to delta oscillations has been added to line 13. In addition, in order to acknowledge that the low frequency is closer to 3 Hz, the manuscript now refers to "delta/theta" instead of just "theta", and a paragraph has been added to the discussion explaining the implications of whether the model frequency best represents delta or theta (lines 304-315).

Could the Authors better explain why dopamin (DA) has a direct excitatory effect on FSIs? Is this supported experimentally? This effect is also important for some of findings: e.g. DA causes the switch of oscillatory activity in FSIs. Is it a model hypothesis? They cite [12] (Methods, line 550; line 33 and 38), however [12] is about modulation of gamma and beta oscillations in hippocampus and there's no mention of neither dopamin nor the striatum.

- This is not a model hypothesis; we put the wrong Bracci et al. paper in the bibliography! The correct citation is Bracci et al. 2002, “Dopamine Excites Fast-Spiking Interneurons in the Striatum”. Thank you for catching that! Additionally, the text of lines 41-42 has been edited to emphasize that this excitation is a result of depolarization from a D1-like receptor.

Figure 2 caption: "I_D" has not been introduced before

- The caption has been edited to clarify that I_D is the D current.

Figure 2:

- Why the orange line starts only at x = 6 and not at x=0?

- It starts there to illustrate that the cell does not fire below a minimum of 40 Hz, whereas when the D-current is not present, the cell has no minimum firing rate. The caption has been edited to reflect this and the figure has been modified to demonstrate this more clearly.

- In C the axis labels and the legend are a bit difficult interpret: blue line is the frequency of theta oscillations and orange is the power according to the legend, why is there something different written on the axis label?

- The axis labels have been changed to match the legend.

Could the authors explain how was the connection probability between FSIs chosen? If there is no experimental data supporting the value of 0.3, it should mentioned among the hypothesis of the model or potential limitations.

- The original connection probabilities were somewhat arbitrary, so we have adjusted the connectivity probabilities to better match experimental data and rerun the simulations accordingly. Fortunately, revising the connection probabilities did not qualitatively change the results. The probability of gap junctions between FSIs is 30% after Hjorth et al, based on Koos and Tepper 1999 and Tepper et al 2004. Gittis et al 2010 found an inhibitory connection probability of 58% between FSIs; we have updated our simulations to match this. FSI to MSN connection probability is now 37.5%, after Corbit et al 2016. The methods section has been updated to include these details (lines 622-629).

Figure 4:

- It would be useful to have a legend in panel A. It could be somewhat understood that the arrow represents DA, etc...but a legend would make it clearer.

- A legend has been added to the panel.

- Also panel D would benefit from a legend. What is the shade? Is it the standard deviation of the average power spectrum of the cells? Why there is no shade in Fig4 Dii? Is this explained at lines 137-138?

- The caption has been updated to explain that the shading represents standard deviation of the mean of 20 simulations, for individual cells separately and the network. An explanation has been added to the text of lines 151-156. Additionally, the visibility of the shading has been enhanced such that it is now clearly visible in Figure 4Dii.

Lines 128-130: this is probably more clear in Fig. 4Di, not 4Ei

- Agreed; the figure reference (now line 153) now mentions both.

The Authors states that DA increases the tonic excitation in the spiny projection neuron, type D1 (SPNs D1), while it decreases tonic excitation in SPNs D2. Why in Figs. 5Ai and 5Aii, 6Ai and6Aii the arrows to SPNs are inverted in the low and high DA conditions? As above, a legend would help.

- The bolded arrows are intended to indicate excitation while the faint arrows are intended to indicate a decrease in excitation. A legend has been added to the schematic clarifying this.

Line 179: this sentence is incomplete.

- The sentence (now lines 198-200) has been reworded with better grammar.

The Authors found that in the network model with SPNs and FSIs, FSIs generate low gamma oscillations (line 184). This result is supported by Fig.1, Fig.2, Fig.4. However these figures refer to models with only FSIs. In Fig. 5iii and 5iv, where the full network (FSIs and SPNs) is shown, there is no clear evidence of low gamma oscillations. The only evidence is after applying wavelet-filtering to the LFP surrogate (Fig.6iD). Could the Authors explain in more detail the analysis? The data shown in Fig.6 seem to be the average of the whole network activity. Do the Authors think that the behavior of isolated FSIs networks are maintained in the full network with SPNs?

- Sorry for the confusion; the spectrograms in figure 5 only show the activity of the SPN subnetworks, which do not express gamma. The gamma is only present in the full network with both FSIs and SPNs. The schematic has been revised to emphasize this and a sentence has been added to the text noting that gamma is not present in the SPN subnetwork. (lines 204-205)

Line 320: "BG" acronym has not been introduced before.

- “Basal ganglia” is now spelled out. (now line 381)

Line 364: "regieme" should be "regime".

- Fixed. (now line 433)

Reviewer #2: The Authors present the analysis of the results obtained simulating biophysical models of the striatal single neurons and microcircuit. In particular, they have observed the evolution of oscillation frequencies of the generated Local Field Potential (when analyzing a population) or membrane voltage (when analyzing a single neuron), at different frequency bands (delta, theta, beta, low/high gamma).

This work focused on the effect of Dopamine levels on the frequency behaviour of the striatal microcircuit; its main contribution is the mechanistic explanation, by means of biophysical neural models, of the theta-modulation of striatal activity caused by high levels of Dopamine. This can justify the observed misbehaviours in motor program execution in pathological conditions, where Dopamine is impacted (e.g., Parkinson's Disease)

The modelling work is sound and well explained, the Authors have organized the manuscript in a way that guides the reader from the single neuron dynamics to the network-level dynamics, explaining at each step the causal-effect mechanisms. The claims are supported by the data presented and are novel, at the same time founded on previous experimental and computational studies on basal ganglia. Relevant literature has been appropriately included in the construction of the model and in the discussion of the results.

In addition to some minor comments, reported below, I just have three concerns:

1) The model of the FSI - D1 SPN - D2 SPN striatal network has been built using 100 neurons per population. However, SPNs make up 95% of striatal neurons in rodents, while interneurons consist of at most 5% of striatal cells. The different ratios in the number of neurons used to build the model and the ones found in the biological network are very different. The Authors addresses this point only in the Methods section (from Line 511). However, this evident discrepancy, given its importance, should be highlighted in the "Caveats and Limitations" section. The Authors should discuss what differences do they expect in the network oscillations with a more biologically-realistic ratio between the number of FSIs and SPNs.

- In order to make the ratio of cells in the network more realistic, we have reduced the number of FSIs in the network to 50 and rerun the simulations accordingly. The network is now 20% FSIs and 80% SPNs, which is biologically plausible in the human striatum (Graveland et al 1985). A paragraph has been added to the “Caveats and limitations” section describing what impact we expect population size ratio to have on the qualitative behavior of the network. (lines 477-488)

2) The figures could be improved and reorganized in order to facilitate the reader. In the manuscript body, figures are referenced not sequentially and, in some cases, the information regarding the same statement is scattered between different figures (e.g., Line 71: Fig. 1B & 2C; Lines 111-112 Fig. Aii referenced before Fig.Ai; Line 114: Fig. 3Cii referenced before Fig.3B; etc.). It would also be useful to split very large graphics like Fig. 5 and Fig. 6 into smaller and more manageable pieces of information, thus increasing the font size of legends and captions, to improve their readability. Other comments on specific figure improvements are reported below.

- All figures have been revised to match the order of the text. Figure 5 has been split up for readability and panel A has been removed from Figure 6. A legend and table of values representing low and high dopamine have been added to the schematics for figures 4 and 5.

3) The Authors have provided a GitHub repository with the codes necessary to run simulations with DynaSim and to reproduce the figures. However, the repository has to be better curated, since it is missing instructions to run the simulations and other details (e.g., what are the versions of MATLAB and the toolbox used). Besides, the codes cannot run since there are paths specified for the developer's machine). I suggest to: i) improve the usability of the provided material ii) use a persistent repository (ModelDB, Harvard Dataverse, etc.) instead of GitHub, to guarantee the preservation and accessibility of the files in the future.

The Data Availability statement should be updated accordingly. Currently, it refers to "Supporting Information files" that do not exist and " Specific data files used to generate the instances of the simulations used for the figures in the manuscript are available upon request" that would not be necessary.

- A readme file describing how to run the simulations and additional documentation have been added to the repository, as mentioned above. In addition, version information has been included in the readme and paths referring to the developer’s machine have been removed. The model has been submitted to modelDB and is pending approval to be made public. The Data Availability statement has been updated to reflect these revisions.

Apart from these three issues, I think the study could make an excellent contribution to the literature, and it fits well within the scope of PLOS Computational Biology, providing sound results to both computational and experimental neuroscience communities studying basal ganglia.

Minor issues:

- In general, please use the conventional space before the unity of measurements in the text (e.g., Fig. 3 Legend, Lines 110, 452, 458, 459, etc.)

- These typesetting errors have been corrected.

- Abstract. Avoid acronyms if possible, to improve its readability.

- Acronyms in the abstract have been written out, with the exception of names of cell types because they are long and used multiple times in the abstract.

- Line 13. Define also the delta band ranges.

- A reference to delta oscillations has been added to line 13; in addition, the low frequency rhythm is now referred to as “delta/theta” throughout the text.

- Figure 1. Additional titles to Panel Ai and Bii could help. (with I_app = X uA/cm2, ... with Poisson noise lambda = Y, etc.). Legends of Aii and Bii are missing the unit of measurement. Please include I_app and g_d in the labels of C and D.

- Figure 1 has been revised with additional labels and legends.

- Figure 1 Legend. For Bii, please specify what do solid lines and shading represent (median and interquartile ranges?) and why this is the only graphics where multiple measurements are presented.

- The caption now specifies that the figure shows mean and standard deviation over 20 simulations. The reason multiple measurements are presented is that the data used to make this subplot had much higher variance than the other subplots in the figure because noise was present in the simulations, while the other plots showed noiseless simulations. This subplot and other plots involving noise have been moved to figure 2.

- Line 74. uA/cm2 is not formatted with mathematical notation

- Fixed. (now line 89)

- Line 86. "the model has no minimum firing rate", this seems to be valid only for I_aa <= 3 uA/cm2. The Authors should also explain why the plot with gd=6 stops at I_app = 3 uA/cm2.

- When gd=6, the neuron does not spike when Iapp < 3 uA/cm2. It’s minimum spiking rate is 40 Hz when Iapp = 3 uA/cm2. In contrast, when the D-current is not present, the spiking rate of the neuron is a continuous function of the applied current. (ie, it rises continuously from zero as soon as sufficient current is applied). The caption has been edited to reflect this. Additionally, lines 7-80 have been edited for clarity.

- Lines 104, 162, 175, 176. The Authors should motivate their choices for the connectivity probabilities. Are there physiology references that they have used to set those values?

- The original connection probabilities were somewhat arbitrary, so we have adjusted the connectivity probabilities to better match experimental data and rerun the simulations accordingly. The probability of gap junctions between FSIs is 30% after Hjorth et al, based on Koos and Tepper 1999 and Tepper et al 2004. Gittis et al 2010 found an inhibitory connection probability of 58% between FSIs. FSI to MSN connection probability is now 37.5%, after Corbit et al 2016. The methods section has been updated to include these details (lines 622-629).

- Line 126. The computation of LFP as the mean voltage of the FSI network is mentioned here for the first time, while it should be introduced at the beginning of the FSI network section.

- This is now mentioned earlier, at the end of the first paragraph of the FSI network section (lines 112-114).

- Figure 3. I suggest inverting panels i and ii, following the logic flow of the manuscript text. This figure has some visualization problems, for example, the blue shading in Aii, B, ii, Ci, and Cii is partially hiding the mean/median blue line.

- The panels have been rearranged and the visualization problems fixed.

- Figure 3 Legend. Please specify what do solid lines and shading represent.

- The caption has been edited to clarify that these represent mean and standard deviation over 10 simulations.

- Figure 4. Panel A. The symbols used for GABA conductance, external excitation, and gap junction conductance should be explained (at least in the legend). The lower-panels E should be clearly marked as insets of higher-panels E.

- Panel A now has a legend and explanation in the caption. The lower panels are now more clearly marked as insets.

- Figure 4 Legend. The Authors should clarify the difference between "FSI population activity" and "all individual voltage traces in the network". Is one of the two the LFP?

- The caption has been clarified, and now reads: Solid line: Power spectral density of summed FSI synaptic currents (surrogate LFP), averaged over 20 simulations. Dashed line: Average power spectral density of each individual FSI voltage trace in the network, averaged over 20 simulations. Shading represents standard deviation from the mean.” The former is the LFP, the latter is a measure from individual cells. This is also described in lines 151-156.

- Line 155. "This previous work", it is unclear what is the reference.

- The reference has been clarified; it refers to Mccarthy et al 2011, “Striatal origin of the pathologic beta oscillations in Parkinson's disease” (now line 170)

- Figure 5. As in Fig. 4, symbols in Panels A should be explained. Panels B are missing indications of the x-axis and y-axis scales and labels. The Authors should add to Panels that the higher and the lower spectrograms come from D1 and D2 populations. Also, the x-axes are missing scales and labels.

- Axis scales and labels have been added; legends have been added specifying which SPN populations the spectrograms come from.

- Figure 6. Panels A have been already shown in Fig. 5. A reorganization of the figures could also solve this kind of repetitions. The labels of Panels B, C and D, as well as the legend of Panels D, are too small to be readable.

- Panel A has been removed and the text size for panels B, C, and D has been increased.

- Line 201. Looking at the slow oscillations of beta and gamma powers in Panel Dii, it seems that the main driving frequency is lower than 5 Hz and closer to 3 Hz.

- Thanks for catching this. In order to acknowledge that the low frequency is often closer to 3 Hz, the manuscript now refers to "delta/theta" instead of just "theta". A paragraph has been added to the discussion explaining the implications of whether the model frequency best represents delta or theta (lines 304-315).

- Line 269. e.g., DAergic

- Fixed (now line 298).

- Line 361. Theta should be used as a symbol.

- Fixed (now line 431).

- Line 364. Bursting regime

- Fixed (now line 434).

- References should be double-checked since there are some formatting errors (e.g., [1] with upper case author names, some references with DOIs some without, different formats to state the publication month, etc.)

- Bibliography formatting has been corrected and standardized.

Reviewer #3: This is a very well-written modeling study that demonstrates the functional interaction of several distinct rhythmic dynamics in the thalamus, and the role of dopamine in regulating this complex systems-level interaction.

Single-cell and network simulations at increasing levels of complexity are presented, with the effects of different parameter choices or simulated perturbations clearly demonstrated at each stage. These scenarios are thoughtfully chosen to provide insight into the mechanisms underlying the dynamics. Finally, the experimental grounding for modeling decisions is clearly specified and supported by references when appropriate, or also supported by references to past modeling literature.

General comments:

1. The conclusions regarding the role of the demonstrated high DA state in switching between cell assemblies or motor programs would be significantly stronger if this switching behavior was directly demonstrated in some way. The full complexity of generating multiple distinct stable assembly states on top of the existing oscillatory dynamics would of course be well beyond the scope of this work and constitute an entire separate paper. However, perhaps some measures of the sensitivity of the existing network to perturbations during the beta-rhythm inhibition could provide a quantitative demonstration of the concept.

- We appreciate this comment as this is exactly the focus of our current work. We agree it would be nice to have a simple demonstration of this phenomenon in this current paper to support our major conjecture about FSIs mediating switching. We have added a supplementary figure demonstrating that the SPN network is more sensitive to perturbation when the beta rhythm is being disrupted by the FSI-induced theta; see Figure S2 and lines 219-221 and 351-355.

2. A bit more information on the choice of network-level parameters in the model would be helpful. The discussion of the D-current conductance in “Caveats and limitations” is quite comprehensive and useful, but a bit of the same logic for how the parameters for effects of dopamine were tuned in the model would be helpful. Did the magnitude and balance of effects on DA receptors and gap junctional conductance need to be tuned to create the phenomena of interest? Such hand-tuning should not detract from the conclusions presented, but a few sentences explaining the approach would be helpful for interpretation and to guide future modeling work.

- The range of applied current was chosen to maintain physiologically realistic firing rates, taken from Berke 2004. Parameters for the magnitude of connections were chosen in order to best demonstrate the behavioral range of the network. Figure 3 shows the inflection points where network behavior qualitatively changes. We have expanded our Methods section to include a more thorough explanation of our choice of dopamine-dependent parameters; please see lines 610-650. We have also included in the main text (lines 457-460) a statement that indicates why we chose the parameters we do, and references the corresponding Methods section.

3. A note on the possible effects of parameter heterogeneity on the phenomena shown would be helpful, especially in the context of the FSI network theta rhythm robustness. Does the robustness to noise conferred by network gap junction coupling hold up even if when there is heterogeneity in the intrinsic properties of the cells?

- We have added a supplementary figure (Figure S1) demonstrating that noise and heterogeneity in the network do not qualitatively impact the network rhythms. This figure is discussed in lines 120-123 and 128-129.

4. The authors mention that the model files are publicly available, but I was unable to locate them within the DynaSim github page. A more direct reference should be provided before publication.

- A readme file containing specific instructions on locating and running the files has been added to the repository. We would appreciate feedback on whether any additional difficulties remain in accessing or running the files, as our goal is to make them easily accessible to the reader.

5. In Figure 5, the rhythmicity of the SPN network looks quite similar between i and iii, as if the frequency preference mechanism for the low DA spiking is present in the subthreshold dynamics as well (although the frequency of the peak is slightly shifted between the subthreshold case in i and the spiking case in iii). This also means that the beta peak is significantly shifted between iii and iv. If the beta dynamics are significantly different between the low and high DA cases (in addition to the theta coupling shown), does this have other consequences for our interpretation of this state?

- The mechanism of subthreshold beta is referenced in lines 185-186. A paragraph has been added to the discussion section about the different roles of low and high beta in the motor system and Parkinson's disease, and how this impacts interpretation of our model (lines 318-330 and lines 408-414)

Minor points:

L66: commas around “stuttering and ga)mma resonance”

- Fixed (now line 68).

L126: in some spots the term “LFP surrogates” is used to convey the fact that this mean voltage is a strong simplification of LFP. That should be mentioned here, where it is first introduced

- An explanation of this term has been added to the first paragraph of the section (lines 112-114).

L171-176: A cross-reference to the “Caveats and limitations” section would be useful to the reader when introducing some of the stronger assumptions of the network model

- The cross-reference has been added (lines 181-183).
